# Valorization of Peanut Skin as Agricultural Waste Using Various Extraction Methods: A Review

**DOI:** 10.3390/molecules28114325

**Published:** 2023-05-25

**Authors:** Nicky Rahmana Putra, Dwila Nur Rizkiyah, Mohd Azizi Che Yunus, Ahmad Hazim Abdul Aziz, Ahmad Shah Hizam Md Yasir, Irianto Irianto, Jumakir Jumakir, Waluyo Waluyo, Suparwoto Suparwoto, Lailatul Qomariyah

**Affiliations:** 1 Centre of Lipid Engineering and Applied Research (CLEAR), Ibnu Sina Institute for Scientific and Industrial Research, Universiti Teknologi Malaysia, Johor Bahru 81310, Malaysia; 2 Faculty of Food Science and Nutrition, Universiti Malaysia Sabah, Kota Kinabalu 88400, Malaysia; 3 Faculty of Resilience, Rabdan Academy, Abu Dhabi P.O. Box 114646, United Arab Emirates; 4 National Research and Innovation Agency, Jakarta 10110, Indonesia; 5 Department of Industrial Chemical Engineering, Institut Teknologi Sepuluh Nopember, Surabaya 60111, Indonesia

**Keywords:** peanut skin, agricultural waste, catechin, antioxidant, green extraction

## Abstract

Peanuts (*Arachis hypogea*) can be made into various products, from oil to butter to roasted snack peanuts and candies, all from the kernels. However, the skin is usually thrown away, used as cheap animal feed, or as one of the ingredients in plant fertilizer due to its little value on the market. For the past ten years, studies have been conducted to determine the full extent of the skin’s bioactive substance repertoire and its powerful antioxidant potential. Alternatively, researchers reported that peanut skin could be used and be profitable in a less-intensive extraction technique. Therefore, this review explores the conventional and green extraction of peanut oil, peanut production, peanut physicochemical characteristics, antioxidant activity, and the prospects of valorization of peanut skin. The significance of the valorization of peanut skin is that it contains high antioxidant capacity, catechin, epicatechin resveratrol, and procyanidins, which are also advantageous. It could be exploited in sustainable extraction, notably in the pharmaceutical industries.

## 1. Introduction

**Figure 1:**
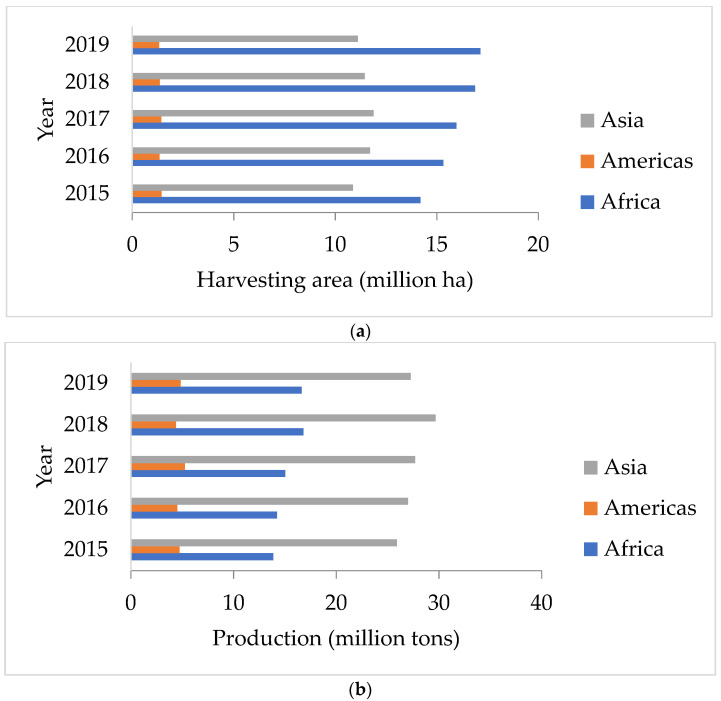
(**a**) Harvest area and (**b**) production of peanuts in Asia, Americas, and Africa [1].

Peanuts are vital crops for the food sector in the world. Peanuts are extensively cultivated in tropical and subtropical locations, and the crop has the ability to commercially advantage both food producers and industrial manufacturers. The average annual worldwide peanut output is 46 million tons, according to studies. As shown in Figure 1b, the Asian regions have the largest harvesting areas and yields compared with America and Africa [1].

The majority of peanuts are processed to manufacture butter, oil, roasted peanuts, sweets, and desserts. During peanut manufacturing, substantial amounts of potentially polluting peanut skin byproducts are produced. Nevertheless, peanut skin is employed as animal feed and fertilizer after its reutilization. Significant quantities of peanut powder, shells, hulls, and vines are also classified as agricultural waste. Due to the fact that the great majority of studies concentrate on oil and kernel production, peanut byproducts such as peanut skin get little study [2].

Moreover, peanut skins are beneficial as they are rich in antioxidants, catechin, epicatechin, resveratrol, and anthocyanidins [3,4]. They may reduce the rate of free-radical-induced oxidation processes [5]. Antioxidants are able to neutralize and build stable compounds by donating additional hydrogen electrons to free radicals [6]. Previous research demonstrated that long-term consumption of peanut skin extract rich in plant polyphenols protects against cancer, cardiovascular disease, diabetes, osteoporosis, and neurological disorders [7,8]. Peanut skin also contains cellulose (40.5%), lignin (26.4%), and hemicellulose (14.7%) [9].

Catechin is the bioactive substance in the peanut skin, which accounts for 17 mg of catechin per g sample **[10]**. Catechin is commonly found in onions, chocolate, wine, grape skin, and tea [11,12,13,14]. It is a flavanol with five attached hydroxyl groups, making it a polar flavanol. It also has been identified as an effective antioxidant. Regarding antioxidant activity, catechin ranks second among the other antioxidants after quercetin [7]. Moreover, catechin is well-known for its many health benefits, which include anti-inflammatory, anti-HIV, antidepressant, and anti-hypertension properties. Therefore, it must be transformed from a zero-value material into a high-value product.

Resveratrol, a stilbene molecule that belongs to the polyphenol family, is often isolated from a wide variety of natural plants, especially from peanut skin. Due to its numerous positive characteristics, resveratrol is extensively used in the culinary and pharmaceutical industries. It is sensitive to structural deterioration and may involve chemical transformations during food preparation [15]. Several studies have consequently focused further on the different elements of resveratrol, such as its anti-aging, antioxidant, and anti-cancer properties [16].

Previously, the bioactive compounds of peanut skin were extracted by traditional techniques such as maceration and Soxhlet. However, the “green extraction” of plant materials has become a challenge for specific industrial experts. This method could provide a higher yield and quality extract with short extraction time, whilst being safe for human health. This is because the extract is free from toxic residues and organic solvents [17,18]. Thus, significant advancements in green extraction, such as microwave-assisted extraction (MAE) and supercritical carbon dioxide (ScCO_2_) extraction, have been introduced [19,20,21,22,23,24,25,26,27]. The extraction method has been crucial to ensuring the end product’s excellent function. Even if the traditional approach provided a larger yield, the long-term repercussions should be addressed, especially in terms of the environment and our health.

The fact that peanut skin includes high levels of antioxidants, catechin, epicatechin, and resveratrol, is also helpful and might be utilized in sustainable extraction processes, especially in the pharmaceutical sectors. Therefore, this article will provide an overview of a recent extraction to valorize the waste peanut skin into high-value products. Some of the optimization methods are briefly described to valorize peanut skin. The published research findings on peanut skin’s physicochemical qualities, antioxidant activity, and phenolic content were examined in greater depth.

## 2. Physicochemical Properties of Peanut Skin

**Figure 2:**
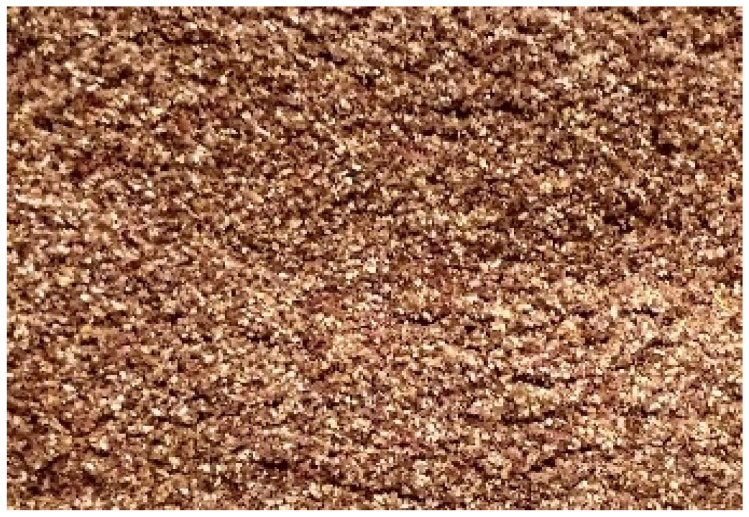
Peanut skin.

Peanut skin, also known as peanut coat, is a common byproduct of peanut factories, as most peanut butter manufacturers remove the seed’s skin (Figure 2). An astringent peanut peel will diminish the flavor of peanut butter. Despite this, peanut skin contains several bioactive components and antioxidants that promote and protect human health, such as phenolic acid, procyanidin, and catechin, to name a few. The skin of non-defatted peanuts contains 90–125 mg/gram of total phenolics, which include phenolic acids, flavonoids, and resveratrol [28].

Soxhlet extraction is frequently used to extract peanut skin, as shown in Table 1. In each gram of dry skin, peanut skin has 140–150 mg of phenolic compounds [29]. The solvents of ethanol, methanol, and hexane were used to extract the phenolic compounds from peanut skin [28,30,31]. Prior studies also found that peanut skin contains 88% of the total antioxidant activity and 30% of the oil extract. Catechin, a bioactive compound, is also highly found in both green tea (17 mg/100 g) and peanut skin (16.1 mg/100 g) [32].

## 3. Bioactive Compounds in Peanut Skin

### 3.1. Catechin

**Figure 3:**
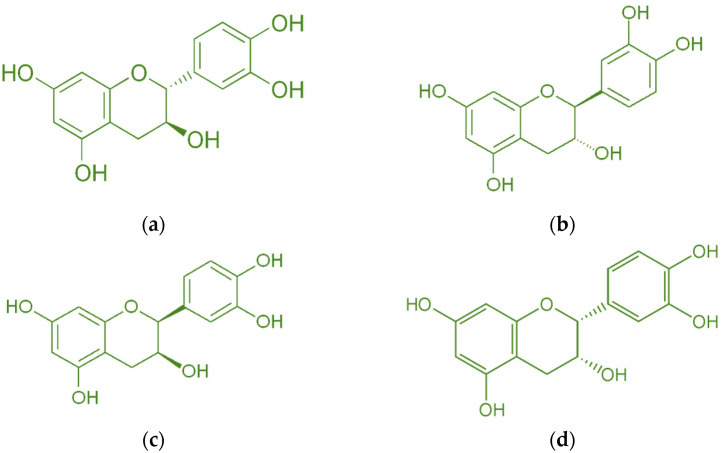
Diastereoisomers of catechin (**a**) (+)-catechin, (**b**) (−)-catechin, (**c**) (+)-epicatechin, and (**d**) (−)-epicatechin.

Catechin is a bioactive molecule found in peanut skin, but it can also be found in tea [34], grape skin [13], wheat [35], onion, apple skin [36], broccoli [12], cocoa [37], and red wine [38]. Catechin has a molecular weight of 290.26 g/mol and a melting point of 175 °C [39]. A high melting point makes it resistant to deterioration at high temperatures. Figure 3 depicts the catechin’s isomer. The most common isomer of catechin is (+)-catechin. The opposite stereoisomer is (−)-catechin, often called ent-catechin. Other names for the most common epicatechin isomer (2R,3R)-(−)-epicatechin are l-epicatechin, epicatechol, (−)-epicatechol, l-acacatechin, l-epicatechol, epi-catechin, 2,3-cis-epicatechin, or (−)-epicatechin. (+)-catechin is thought to exert pharmacological effects on the human body, such as cardioprotective, diuretic, and hypotensive effects [11]. Reactive oxygen species (ROS) and hydroxyl radicals have the potential to cause oxidative damage to bodily cells. Catechin, on the other hand, possesses antioxidant qualities that prevent this damage [34]. Catechin has been demonstrated to have superior antioxidant qualities and a greater capacity to scavenge free radicals than vitamin C, E, or -carotene carotene [40,41,42,43]. Moreover, catechins can transfer hydrogens through their hydroxyl groups.

Catechin belongs to the favan-3-ol family. Regular use can help prevent Parkinson’s, diabetes, cancer, and other diseases [44]. However, producing highly active catechin may be problematic because it is rapidly broken down when exposed to light due to UV radiation from the sun [2,45]. Furthermore, catechin reduces hemoglobin levels by delaying the oxidative process in hemoglobin cells [46]. Katalinić, et al. [33] used a bioassay to determine the antioxidant activity of many well-known antioxidants as shown in Table 1. Furthermore, Table 2 shows that various materials contain catechin as a bioactive compound [32]. The catechin content of peanut skin is comparable to that of green tea and higher than that of black tea [14]. Peanut skin contains a high concentration of catechin, prompting the development of peanut skin as a catechin resource to replace other catechin resources such as green and black tea.

### 3.2. Resveratrol

**Figure 4:**
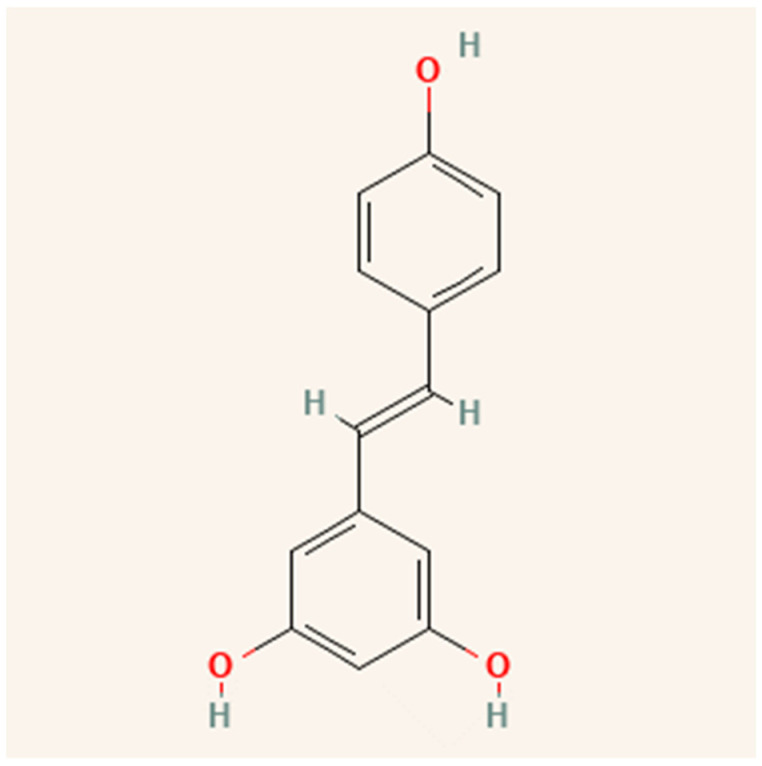
Structure of resveratrol.

Resveratrol (3,4′,5-trihydroxystilbene) is a stilbene, a type of polyphenolic compound. Certain plants produce resveratrol and other stilbenes in response to stress, injury, fungal infection, or ultraviolet (UV) radiation [49]. As shown in Figure 4, resveratrol is a fat-soluble molecule with trans and cis molecular configurations [50]. Both cis- and trans-resveratrol occur as glucose-bound glucosides [51]. Romero-Pérez, et al. [52] discovered that resveratrol-3-O-glucoside, also known as piceid, is one of the most important resveratrol derivatives. Resveratrol can be found in grapes [53], wine [54], grape juice [55], peanuts [15], cocoa [56], and berries [57].

Researchers worldwide have investigated resveratrol’s health consequences since the early 1990s, when the presence of resveratrol in red wine was proven [58]. It was hypothesized, in particular, that moderate red wine consumption containing resveratrol could help explain why the French have a relatively low incidence of coronary heart disease (CHD) despite eating foods high in saturated fat, a phenomenon known as the “French Paradox” (cardiovascular disease) [59]. Since then, scientific interest has grown in resveratrol’s ability to prevent cancer, delay the onset of cardiovascular and neurological diseases, improve glycemic control in type 2 diabetes, and lengthen lifespan in experimental animals.

Resveratrol has been extracted from peanut skin using ethanol with the maceration method [29]. On the other hand, this technique has limitations including long extraction time and excessive solvent usage. Therefore, resveratrol was recently extracted from peanut skin using modern techniques such as MAE [30]. Modern extraction techniques and environmentally friendly solvents have been developed to extract resveratrol efficiently and effectively [10]. Ionic liquids (ILs) combined with MAE [60] are categorized as green solvents and are also used to extract resveratrol. This method has a high dissolution rate and absorbs more microwave radiation than organic solvents [61].

### 3.3. Procyanidins

**Figure 5:**
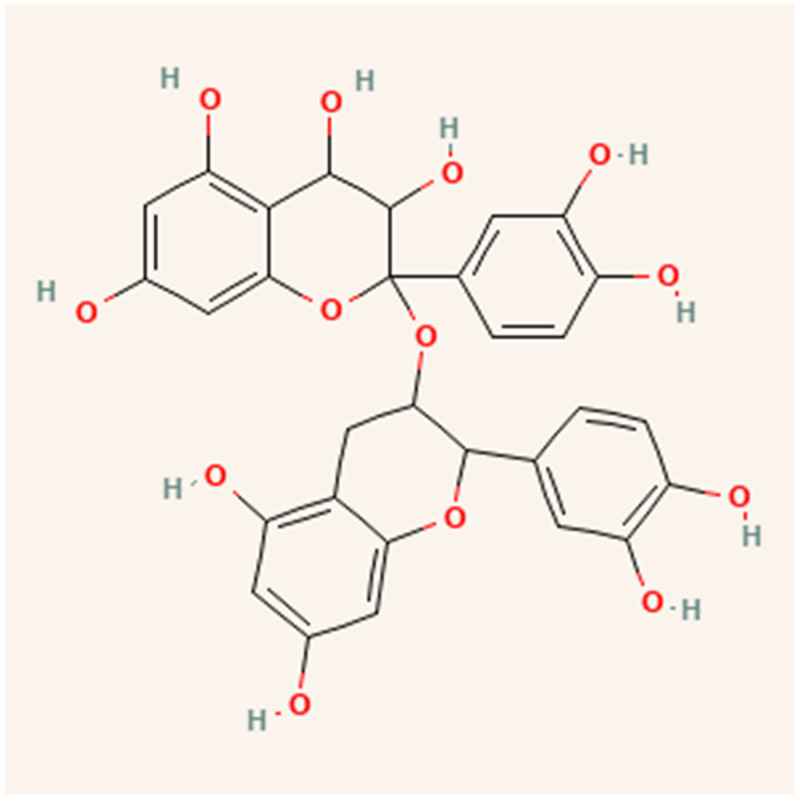
Structure of procyanidins.

Many agro-industrial wastes from the food processing sectors, including cocoa, berries, grapes, apples, blueberries, plums, tea leaves, coffee, cinnamon, peanuts, and leguminous plants, include procyanidins and their monomers [62]. Depending on the origin and kind of the plant material, the procyanidin concentration may vary [60]. These molecules contain flavan-3-ol monomers as basic units in their structure, which are composed exclusively of (+) catechin and (–) epicatechin. (Epi)catechin monomers may be biosynthetic precursors of procyanidins [63] as shown in Figure 5.

Sarnoski, et al. [64] found that there are several forms of proanthocyanidins in peanut skins, but A-type proanthocyanidins predominate. Peanut skins are mostly composed of dimeric, trimeric, and tetrameric proanthocyanidins species. Compared to single solvent extractions, the multistep extraction approach is an excellent method for concentrating procyanidins from peanut skins. The optimal parameters for ScCO_2_ extraction were 20.39 MPa, 333.23 K, and 0.17 mL/min for procyanidins and proanthocyanidins containing 2325.23 and 409.95 g/g, respectively [4].

## 4. Antioxidant Activity of Peanut Skin Extract

**Figure 6:**
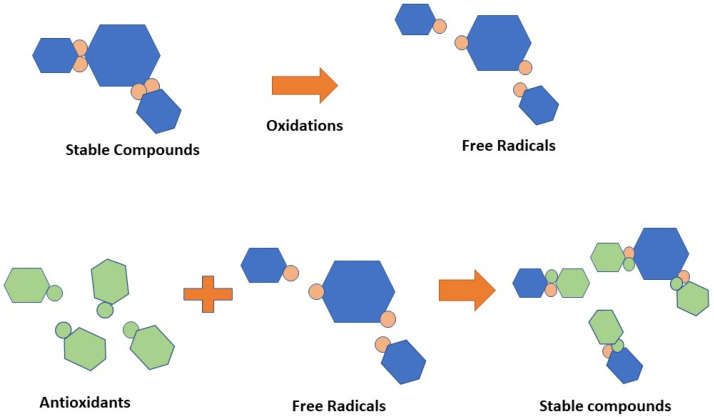
Scheme of antioxidants.

The body requires antioxidants to interact with free radicals and interrupt the chain reaction that leads to sickness [65]. Antioxidants are chemical classes that can hinder oxidation cycles, preventing or delaying the oxidative destruction of biomolecules. The antioxidant mechanism is depicted in Figure 6. Vitamin C, vitamin E, xanthophylls, carotenes, flavonoids, lignans, and stilbenes were the most common antioxidant-active compounds in the peanut skin [6,66,67,68,69,70].

The free radical 2,2-diphenyl-1-picrylhydrazyl (DPPH) is commonly used to test compounds’ ability to function as free radical scavengers and assess the antioxidant activity of antioxidant components [71]. The DPPH approach, which applies to the overall antioxidant capacity of the sample rather than any specific antioxidant component, can be employed for solid or liquid samples [72]. A measure of total antioxidant capacity will help us understand the antioxidant compound’s functional properties [73]. As indicated in Table 3, several researchers have successfully used the DPPH method to assess antioxidant activity. Table 4 shows that peanut skin offers higher antioxidant activity compared with several sources of antioxidant compounds. As a result, peanut skin can be used to replace renewable or sustainable sources in health and wellness goods.

## 5. Valorization of Peanut Skin by Conventional and Green Extraction

Extraction is commonly defined as separating phytochemicals from plant components [84]. Plant materials differ in phenolic chemicals, flavonoid compounds, and tannins [85,86,87]. Different extraction conditions are required to obtain maximum extract yield recovery and excellent extract quality [3,4,7,78,88,89]. An extract’s number of bioactive compounds is critical for assessing its quality [90]. Many factors influence the efficiency and quality of bioactive chemical extraction, including the type of extraction solvent, solvent concentration, extraction temperature, extraction pH, and extraction duration [91]. There are two extraction processes based on overall separation: conventional and green. Soxhlet extraction is categorized as a conventional extraction method and is commonly applied to extract plants and herbs [92]. It uses a toxic solvent including methanol, n-hexane, and other toxic solvents. ScCO_2_, MAE, and UAE are categorized as green extraction methods due to possessing non-toxic solvents, shorter extraction times, low energy consumption, and providing higher quality extracts [90]. The previous studies of peanut skin extraction are shown in Table 4.

### 5.1. Soxhlet Extraction on Peanut Skin Valorization

In 1879, Von Soxhlet invented a novel extraction process, which became the most widely used leaching technology for an extended period. It is always a critical metric of success against which novel leaching strategies are measured. The benefits and drawbacks of this extraction process have been exploited to generate various changes to alleviate or reduce the extraction time while enhancing the quantity and quality of extract. This technique requires fewer minor procedures, can extract more sample mass, and appears devoid of matrix effects [93,94,95].

Ju and Howard [96] optimized the Soxhlet extraction conditions for maximum peanut skin recovery. Ethanol proportions (0 to 96% *v*/*v*), particle sizes (0 to 10 mm and non-crushed skin), and solid–liquid proposition (20 to 60 mL/g) were used in the extraction. The results showed that 70% ethanol, non-crushed peanut skin, and a solvent/solid ratio of 20 mL/g were the best conditions, yielding a maximum yield of 0.118 g/g. The solid–liquid solvent and ethanol concentration ratio were significant factors in increasing yield recovery.

Methanol, ethanol, and water were used as solvents, with concentrations ranging from 0 to 90%, temperatures ranging from 30 to 60 °C, and extraction times ranging from 10 to 30 min. The responses were TPC, ORAC level, and resveratrol content. TPC was highest in ethanol extracts, followed by methanol and water. The maximum TPC predicted was 118 mg/g. The highest ORAC activity was found in methanol extracts, which had 2149 mol of TE/g, followed by ethanol and water [81]. Nepote [96] also reported that ethanol is the best solvent for extracting peanut skin via Soxhlet extraction. This is because ethanol can extract both polar and nonpolar compounds. As a result, all bioactive compounds found in peanut skin can be extracted effectively [31].

### 5.2. MAE on Peanut Skin Valorization

The frequency range of microwaves is 300 MHz to 300 GHz [97]. In contrast to the traditional way, thermal energy is wasted to the environment. The heating in MAE is selective and localized. This new heating method might greatly shorten extraction time [98]. Microwave heating is dependent on polar solvent contact, which is determined by ionic conduction and dipole rotation [92,97,99]. Ionic conduction refers to the electrophoretic transport of ions in a variable electromagnetic current. The resistance of the solution to ion movement generates friction, which warms the liquid. Rotation of the dipoles realigns them with a rapidly changing electromagnetic current [100]. In one to two minutes, the radiation may hydrolyze the ether bonds of cellulose; thus, the component of plant cell walls becomes soluble into the solvents. High temperature promotes cellulose dehydration and decreases its mechanical strength in the cell wall enabling the solvent to more effectively promote the solubilization of compounds into the solvent [101].

Solvent selection in MAE for the valorization of peanut skin is influenced by the target analyte’s solubility, the solvent’s penetration, interaction with the sample matrix, and the dielectric constant [102]. Ethanol, methanol, and acetone were used to extract phenolic components from peanut skin, yielding a higher yield of polyphenols than ethanol extraction, despite the latter extract having superior antioxidant capabilities [103].

Ballard, Mallikarjunan, Zhou and O’Keefe [30] investigated the effects of microwave power (10% to 90%), irradiation time (30 to 150 s), and sample mass (1.5 to 3.5 g) on TPC and ORAC levels of peanut skin extracts. The optimized conditions of 90% power, 30 s of irradiation time, and 1.5 g of skins yielded a maximum TPC of 143.6 mg/g and an ORAC level of 2789 mol/g. Higher microwave power with shorter irradiation time was the best combination for obtaining high TPC and ORAC levels. Peanut skin pores will be broken by high microwave power. As a result, ethanol will easily penetrate the pores of peanut skin as a solvent. Therefore, the extract will be more soluble in the solvent and have less mass transfer resistance. Higher microwave power also increases solvent diffusivity [104]. The disadvantage of irradiation duration could not be ignored because exposing the compounds to microwave irradiation for an extended time decomposes the monomeric catechin and reduces efficiency [105].

Bai, et al. [106] discovered that microwave extraction had a higher extraction rate in total flavonoid recovery from peanut skin. The optimal extraction conditions were a microwave power of 690 W, an extraction time of 40 s, an ethanol concentration of 55%, and a material–liquid ratio of 1:20. The maximum total flavonoid yield was 3.18%. The simple microwave extraction method can quickly extract total flavonoids from peanut skin. The extraction rate will increase as the microwave power increases. Localized heating occurs in the sample because of microwave power. It causes MAE to degrade the plant matrix, allowing the extract to diffuse and dissolve in the solvent.

Meanwhile, increasing the power enhances the extraction yield and allows for a shorter irradiation time [107]. However, high microwave power may result in low extraction yields due to the destruction of thermally sensitive compounds. Increasing microwave power improves extraction yield until it becomes minor or declines [108]. A good solvent-to-solid ratio ensures homogeneous and efficient heating. Meanwhile, excessive solvent results in poor microwave heating because the solvent absorbs microwave radiation, necessitating more power. Since active compounds are concentrated in specific areas, a low solvent-to-solid ratio creates mass transfer barriers, limiting compound transport out of the cell matrix [109].

### 5.3. UAE on Peanut Skin Valorization

UAE is less costly, quicker, and more versatile than earlier techniques since it can employ solvents with varying polarity. Despite its benefits, this method has difficulty integrating numerous instruments and automation [110,111]. Gharibzahedi, et al. [112] discovered that ultrasonic frequency improves extraction yields more efficiently than the previous extraction method. Additionally, UAE extracts were less cytotoxic than solvent and hydrothermal extraction techniques. Irradiation also led to matrix disintegration, but ultrasonic waves promoted matrix hydration [113].

Jin, Gao, Kong, Yang, Kuang, Yang, Fu, Cheng and Li [10] developed an improved and sustainable technique for the extraction of resveratrol from peanut skin using microbial consortiums immobilized on cellulose and an ultrasound-assisted surfactant aqueous system pretreatment. The best immobilized microbial consortium on cellulose was formed by *Aspergillus oryzae* and *Aspergillus niger* yeast. Other ideal conditions included 3% Triton X-114, a liquid–solid ratio of 25:1, an ultrasonic power of 200 W, a culture temperature of 30 °C, and a culture period of 36 h. The study found that resveratrol concentration reached 96.58 g/g under these conditions.

The suggested pretreatment approach employing microbial consortiums immobilized on cellulose with ultrasound-assisted surfactant aqueous solution for the extraction and bioconversion of target chemicals from plant materials proved to be efficient, rapid, environmentally friendly, and inexpensive. As a result, the method described in this article could be a viable and efficient way of extracting resveratrol from peanut skin. It also could be widely used to produce targeted compounds from plant waste residue. Syahdi, et al. [114] discovered that many conditions were optimized for resveratrol, including solvent types with 70% ethanol and natural deep eutectic solvent (NADES). The results showed that a 1:20 solid-to-liquid NADES ratio and a 15 min extraction time provided more resveratrol (0.049 mg/g) than 70% ethanol (0.011 mg/g).

### 5.4. ScCO_2_ Extraction on Peanut Skin Valorization

ScCO_2_ is applied when the fluid condition exceeds the critical temperature of 31.1 °C and a pressure of 7.1 MPa. ScCO_2_ is advantageous due to its high diffusivity and broad density range. It depends on the solubility of the solvent. ScCO_2_ has a density, viscosity, and diffusivity that are intermediate between a gas and a liquid. The critical temperature is the highest temperature at which a gas can transform into a liquid phase when the pressure is increased. Meanwhile, the critical pressure is the highest pressure at which a liquid can transform into a gas when its temperature increases.

ScCO_2_ extraction of peanut skin has previously been studied by Putra, Rizkiyah, Zaini, Machmudah and Yunus [78]. Initially, they discovered that the best conditions were 0.17 mL/min, 21.86 MPa, and 332.23 K, with 3399.84 g/g of epicatechin and 752.03 g/g of catechin. The solubility of epicatechin and catechin was between 6.02 E-5 g/L and 1.21 E-5 g/L. Furthermore, catechin concentration increased with increasing temperature. The high temperature increases the diffusivity of the solvent, allowing it to penetrate the particle pore of the peanut skin. As a result, the catechin dissolves easily in the solvent. High temperatures also reduce the viscosity of carbon dioxide. The viscosity of a lighter solvent increases mass transfer and decreases resistance. Therefore, the higher catechin and epicatechin yield will be increased. A high-pressure environment was also suitable for extracting catechin and epicatechin. This is because the high pressure will increase the density of carbon dioxide, thus, enhancing carbon dioxide’s solvation power.

Moreover, ScCO_2_ could extract high procyanidins and proanthocyanidins as promising compounds from peanut skin [4]. The best conditions were 0.17 mL/min of ethanol, 20.39 MPa, and 333.23 K, with the outcomes of 409.95 g/g of proanthocyanidins and 325.23 g/g of procyanidins. With an average absolute relative deviation of 3.01%, the Chrastil model had the best connection to flavonoid solubility. Pressure and temperature increase the extraction efficiency of ScCO_2_ to recover both procyanidins and proanthocyanidins. As a modifier, ethanol improves the recovery of procyanidins and proanthocyanidins. This is because ethanol increases the polarity of carbon dioxide; thus, combining ethanol and ScCO_2_ may extract polar compounds. ScCO_2_’s only limitation is that it only works with nonpolar compounds and oil extracts. Thus, adding ethanol can overcome this limitation in extracting high polar compounds. Putra, et al. [115] also discovered higher TPC recovery from peanut skin when there were high pressure and high temperature conditions. The optimal conditions were 29.43 MPa, 319.16 K, 0.09 mL/min, and 290.12 mg/L TPC. The results showed that a high temperature condition improves phenolic content solubility while a low temperature environment improves flavonoid solubility.

## 6. Future Perspective of Peanut Skin Valorization

Attention has switched from emission reduction to a more pragmatic approach in waste management [92,99]. Waste products are still viable commodities, which are essential for sustainable growth. The processing, use, and disposal of agro-industrial wastes have made them a worldwide issue. As a consequence, population growth has led to the depletion of natural resources. In the biorefinery concept, “circular economy” has emerged lately. These models aim to use and manage waste from certain industrial processes as sustainable raw materials, with an emphasis on the economic and environmental issues.

Since peanut skin is generated in enormous quantities, it must be processed immediately to prevent putrefaction. Processing waste into goods with added value has the potential to reduce waste and become a feasible solution for the world’s population. It has high antioxidative, antibacterial, and antiviral capabilities that are extremely helpful and might be used in future nutraceutical and pharmaceutical sectors. In the past, extraction techniques, such as maceration and Soxhlet, were often utilized to obtain peanut skin. The “green extraction” of plant material has, however, overtaken the classic extraction method. Green extraction has been encouraged in order to improve production at a lower price. In the absence of an organic solvent, this method may also avoid the production of hazardous residues. As a consequence, significant advances have been achieved in green extraction methods, such as MAE and ScCO_2_.

To meet growing demand, however, the process’s efficiency, predictability, and reproducibility of product quality must be enhanced. As emphasized in this study, microwave, ultrasonic, and ScCO_2_ are among the successful and trustworthy innovative techniques explored for integrating the pectin extraction method, with varied degrees of performance. Even if these processes are quantitatively and qualitatively suitable for laboratory use, a lack of expertise hinders their usual industrial use. They cannot be used for scale-up, since continuous methods are preferable.

Some of these innovations are too costly for new and small producers; however, this may not be the case for bigger specialty chemical/ingredient businesses. However, careful optimization of the process parameters of the most recent techniques is necessary. Eventually, market participants will adopt one or more of these methods to make specialized catechin and resveratrol, most likely with microwave heating for rapid mass transfer. In addition to industrially produced peanut skin, several research institutions and labs have focused on identifying and exploiting peanut skin as a raw material for catechin synthesis. In addition, there is little study on the recovery of catechin/bioactive chemicals from peanut skin using environmentally friendly methods such as UAE and ScCO_2_. However, classic extraction techniques such as Soxhlet and MAE were commonly used. As a consequence, there is a technical gap in the use of environmentally friendly methods to extract this bioactive compound.

## 7. Summary of Various Extraction Methods to Valorize the Peanut Skin

Table 5 compares the benefits and drawbacks of various extraction techniques for valorizing peanut skin. The traditional Soxhlet process yields more but has lower quality than Soxhlet ScCO_2_. MAE outperforms Soxhlet extraction in terms of quality because it uses a shorter extraction time at a lower temperature. The UAE is also improving the Soxhlet extraction process, resulting in less energy consumption, a shorter extraction time, and a higher quality extract. However, ultrasonic waves have been shown to degrade certain phenolic acids and produce highly reactive hydroxyl radicals inside the gas, which are drawbacks of this method. Meanwhile, ScCO_2_ is a simple way to valorize the peanut skin and is categorized as green and sustainable. This is because CO_2_ extracts catechin, resveratrol, and antioxidant compounds from peanut skin, and it is inert and safe for health and wellness products, making it a green solvent. Furthermore, the extraction time is reduced compared to previous techniques such as Soxhlet, MAE, and UAE. Thus, energy consumption can be reduced. The only disadvantage of ScCO_2_ is that it is operated in high pressure conditions. Therefore, this will impact upon higher cost production and safety.

## Figures and Tables

**Table 1:** Antioxidant activity (AA) ranking of various bioactive compounds [33].

Rank	Bioactive Compounds	AA (%)
**1**	Quercetin	95
**2**	Catechin	94
**3**	Trolox	92
**4**	Vitamin E	91
**5**	Butylated Hydroxytoluene (BHT)	49

**Table 2:** Catechin recovery from various materials.

Materials	Catechin (mg/g)	Source
Peanut skin	16.1	[28]
*Areca catechu*	0.0716	[47]
Green tea	17.7	[32]
Grape skin	12	[13]
Black tea	5.6	[14]
Spearmint	0.14	[48]

**Table 3:** Antioxidant activity from another material.

Materials	AA (%)	Reference
Peanut skin	97.70	[28]
*Limnophila aromatica*	88.56	[74]
Green tea	86.05	[69]
Grape skin	91.39	[75]
Black tea	59.34	[69]
*Piper betle* leaves	92.34	[67]
*Quercus infectoria* galls	96.97	[76]

**Table 4:** Previous studies on the valorization of peanut skin.

Source	Results
Nepote, Grosso and Guzman [29]	The best Soxhlet extraction conditions were ethanol 70% and solvent/solid ratio of 1:20 g/mL. The highest yield obtained was 0.12 g/g.
Ballard, Mallikarjunan, Zhou and O’Keefe [30]	The optimum MAE variable was 90% of power, 30 s of irradiation time, and 1.5 g. The highest TPC and ORAC of skins were 143.6 mg/g and 2789 μmol/g, respectively.
Bodoira, et al. [77]	The maximum TPC was achieved using 60.5% ethanol at a temperature of 220 °C and a 7 g/min flow rate.
Putra, Yunus, Ruslan, Idham and Idrus [31]	Soxhlet extraction gave the highest yield (36.22%) using ethanol compared with ScCO_2_ extraction (15.47%) at 30 MPa, 70 °C. The extracts of ScCO_2_ extraction yielded the higher catechin (208.73 µg/g) compared with Soxhlet extraction (42.24 µg/g).
Putra, Rizkiyah, Zaini, Yunus, Machmudah, Idham and Hazwan Ruslan [68]	Mean particle size of 425 µm gave the highest yield extract and antioxidant activity by using ScCO_2_ extraction (15.53% extract, 93.43% antioxidant activity) and Soxhlet extraction (36.28%, 62.21% antioxidant activity).
Putra, et al. [78]	Higher pressure and lower temperature conditions increase the peanut skin oil recovery using ScCO_2_ extraction.
Yu, et al. [79]	Peanut skin was extracted using Soxhlet extraction that contains a highest TPC (125 mg/g and TAA (3.39 mM_TE_/mM).
Ying [80]	The optimum UAE conditions were ethanol of 60%, solid–liquid ratio of 1:8, temperature of 50 °C, and extraction time of 20 min. The maximum yield obtained was 33.25%.
Ballard, et al. [81]	In the extraction of peanut skin using MAE, ethanol gave the highest TPC recovery of 118 mg/g and yield of 30.8% at temperature of 30.9 °C and extraction time of 12 min. However, methanol offered the highest ORAC activity of 2149 μmol/g.
Wu, et al. [82]	The optimum MAE parameters were the solid–solvent ratio of 1:25, ethanol of 75%, extraction time of 2 min, and microwave power of 540 W, with the responses of TPC being 183.25 mg/g.
Braga, et al. [83]	The highest content of total flavonoids was obtained from peanut skin, with 2.44 mg/g compared to grape (1.76 mg/g) and mango (1.70 mg/g).

**Table 5:** Summary of advantages and disadvantages of peanut extraction.

**Methods**	**Benefits**	**Drawbacks**
Soxhlet	Less energy consumption Less quantity solvent Higher yield	Toxic solvent High temperature Long extraction time Low quality of extract
MAE	Shorter extraction time with higher yield extract instead of Soxhlet extraction Less solvent consumption Better quality of extracts compared to Soxhlet	High temperature
UAE	Low energy consumption Fewer extraction times Higher extraction yields No high pressure or temperature High purity of extract	Unsuitable for phenolic recovery
ScCO_2_	Suitable for nonpolar compounds extract Shorter extraction time Safe solvent for health and wellness products Higher quality of extract	High pressure extraction High cost of operation Low extraction efficiency for polar compounds

## Data Availability

Data available on request from the authors.

## Nomenclature

AA	antioxidant activity
AH	antioxidant
CER	constant extraction rate
CHD	coronary heart disease
DPPH	2,2-diphenyl-1-picryl-hydrazyl-hydrate
FER	falling extraction rate
GAE	gallic acid equivalent
IL	ionic liquid
MAE	microwave-assisted extraction
NADES	natural deep eutectic solvent
ORAC	oxygen radical absorbance capacity
R	radical
ROS	reactive oxygen species
ScCO_2_	supercritical carbon dioxide
SWE	subcritical water extraction
TAA	total antioxidant activity
TE	Trolox equivalent
TFC	total flavonoid compounds
TPC	total phenolic compounds
UV	ultraviolet
